# The Luminescent Oligothiophene p-FTAA Converts Toxic Aβ_1–42_ Species into Nontoxic Amyloid Fibers with Altered Properties*

**DOI:** 10.1074/jbc.M115.696229

**Published:** 2016-02-23

**Authors:** Livia Civitelli, Linnea Sandin, Erin Nelson, Sikander Iqbal Khattak, Ann-Christin Brorsson, Katarina Kågedal

**Affiliations:** From ‡ Experimental Pathology, Department of Clinical and Experimental Medicine and; § Division of Molecular Biotechnology, Department of Physics, Chemistry, and Biology, Linköping University, Linköping, Sweden

**Keywords:** aggregation, Alzheimer disease, amyloid-β (AB), cell death, fibril

## Abstract

Aggregation of the amyloid-β peptide (Aβ) in the brain leads to the formation of extracellular amyloid plaques, which is one of the pathological hallmarks of Alzheimer disease (AD). It is a general hypothesis that soluble prefibrillar assemblies of the Aβ peptide, rather than mature amyloid fibrils, cause neuronal dysfunction and memory impairment in AD. Thus, reducing the level of these prefibrillar species by using molecules that can interfere with the Aβ fibrillation pathway may be a valid approach to reduce Aβ cytotoxicity. Luminescent-conjugated oligothiophenes (LCOs) have amyloid binding properties and spectral properties that differ when they bind to protein aggregates with different morphologies and can therefore be used to visualize protein aggregates. In this study, cell toxicity experiments and biophysical studies demonstrated that the LCO p-FTAA was able to reduce the pool of soluble toxic Aβ species in favor of the formation of larger insoluble nontoxic amyloid fibrils, there by counteracting Aβ-mediated cytotoxicity. Moreover, p-FTAA bound to early formed Aβ species and induced a rapid formation of β-sheet structures. These p-FTAA generated amyloid fibrils were less hydrophobic and more resistant to proteolysis by proteinase K. In summary, our data show that p-FTAA promoted the formation of insoluble and stable Aβ species that were nontoxic which indicates that p-FTAA might have therapeutic potential.

## Introduction

Alzheimer disease (AD)^5^ is the most common form of dementia and is characterized by progressive impairment in episodic memory and cognition. Neuropathologically, AD is characterized by the formation of neuritic plaques that are primarily formed by deposits of the amyloid-β peptide (Aβ) and neurofibrillary tangles (NFTs), which are aggregates of the hyperphosphorylated tau protein (1). Aβ is generated from the amyloid precursor protein by sequential proteolytic cleavage of β- and γ-secretases (2). It was proposed that soluble oligomers of Aβ are neurotoxic but that the insoluble amyloid fibrils and plaques within the brain are inert (3, 4). Plaques can occur without clinical features of neurodegeneration, and there is a poor correlation between the presence of plaques and cognitive dysfunction, as demonstrated by studies in AD animal models and in humans (5–7). Instead, the severity of disease progression correlates with Aβ oligomers, which have been found in mouse models of AD and in the cerebrospinal fluid and brain tissue from AD patients (8–10). *In vitro* studies of Aβ aggregation have demonstrated that Aβ monomers convert to small oligomers, which in turn form larger oligomers through a complex process that ultimately leads to the formation of insoluble fibrillar aggregates (11). The aggregation of Aβ is the starting event in a cascade, which culminates in neuronal cell death and memory impairment (12). Thus, it is important to develop powerful tools that can discriminate among the different conformational states of Aβ to understand the role of transient aggregate species in the progression of AD.

Recently, luminescent-conjugated polythiophenes (LCPs) were introduced as a novel class of fluorescent probes for the selective staining of amyloid aggregates (13–15). Thereafter, a second generation of thiophene-based amyloid probes was developed using smaller, hydrophobic LCPs called luminescent-conjugated oligothiophenes (LCOs) (16). LCOs have a flexible conjugated thiophene backbone with a defined length and, depending on both the length of the backbone and the chemical properties of the side chains, a capacity to spectrally discriminate among Aβ deposits, NFTs and dystrophic neurites in brain sections of AD patients (17). Their behavior is different from other canonical dyes, such as Congo red and Thioflavin T (ThT), because LCOs can detect small differences in the structures of protein aggregates due to alterations of the thiophene backbone. These changes in the conformation of the LCOs lead to changes in their fluorescence properties that result in the capacity of the LCOs to discriminate among protein aggregates (18–20). The LCOs p-FTAA (penta-formylthiophene acetic acid) and h-FTAA (hepta-formylthiophene acetic acid) have absorption and emission properties that can be altered according to the different morphologies of protein aggregates (21). The tetrameric LCO q-FTAA (quadro-formylthiophene acetic acid) stains end-stage amyloid fibrils in AD-affected regions of the brain (17). Therefore, it is clear that the chemical composition of LCOs directs their capacity to detect different amyloidogenic structures, thus revealing their versatility in staining distinct aggregates that are formed at different stages during the Aβ aggregation process.

**FIGURE 1.**
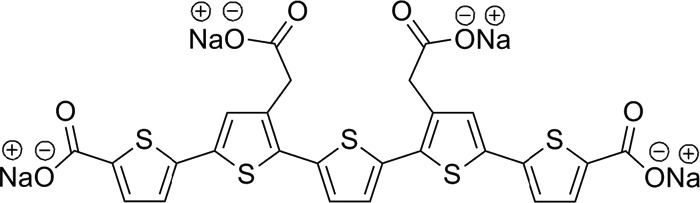
**Chemical structure of the LCO p-FTAA.**

The process of fibrillation can be manipulated by small compounds that bind to Aβ deposits or prefibrillar species. It was demonstrated that ThT reduces the process of Aβ fibrillation in an AD model of *Caenorhabditis elegans,* thereby extending the life span of the worms (22). Resveratrol and (−)-epigallocatechin-3-gallate stabilize oligomers and inhibit Aβ fibrillation, thus preventing the cytotoxicity in cell culture models (23, 24). Tramiprosate is a compound that possesses the ability to inhibit Aβ aggregation to reduce neuronal death (25), and scyllo-inositol is a compound that can rescue memory function in normal adult rats treated with soluble Aβ oligomers by interacting directly with Aβ (26). Compounds that accelerate Aβ fibrillogenesis were also shown to decrease the toxic effect of Aβ, such as curcumin, which extended the lifespan in *Drosophila melanogaster* flies that overexpressed Aβ by accelerating the maturation of Aβ to amyloid fibrils (27). Methylene blue and the orcein-related small molecule O4 also promote the formation of mature Aβ fibrils, thereby preventing cytotoxicity (28, 29). Among the various LCOs, p-FTAA (Fig. 1) is the most characterized molecule and has an antitoxic effect on the prion protein (30, 31). Since p-FTAA binds to Aβ species (17), we explored whether this binding could mediate a change in Aβ toxicity. By using both biochemical and biophysical approaches, we demonstrated that p-FTAA decreased Aβ toxicity via the formation of non-toxic and insoluble fibrillar Aβ species.

## Experimental Procedures

### 

#### 

##### Preparation of the Aβ_1–42_ Peptide and the LCO p-FTAA

Recombinant Aβ_1–42_ (rPeptide) was dissolved in trifluoroacetic acid, which was removed by lyophilization. The peptide was re-dissolved in 1,1,1,3,3,3-hexa-fluoro-2-propanol (HFIP), aliquoted and then lyophilized. The aliquots were kept at −80 °C. Prior to each experiment, the lyophilized Aβ_1–42_ was dissolved in 2 mm NaOH to 222 μm and further diluted in PBS (140 mm NaCl, 2.7 mm KCl, 10 mm, pH 7.4) with or without p-FTAA. The LCO p-FTAA was synthesized as described earlier (16).

##### Cell Culture

Human SH-SY5Y neuroblastoma cells (ECACC, Sigma Aldrich) were cultured in Minimum Essential Medium (MEM) Glutamax (Invitrogen), supplemented with 10% fetal calf serum (FCS; PAA Laboratories), 50 units/ml penicillin, 50 μg/ml streptomycin, and 2 mm glutamine (Lonza) and maintained at 37 °C in 5% CO_2_. Prior to each experiment, the cells were differentiated with 10 μm retinoic acid (RA; Sigma Aldrich) for 7 days and were then seeded at a density of 30,000 cells/well in a Corning Costar tissue culture 96-well plate (Corning Inc. Life Sciences).

##### Viability Test

Twenty-four hours after seeding the cells, Aβ_1–42_ was diluted in PBS to a concentration of 30 μm, aggregated with or without p-FTAA (1.5 mm stock solution) and diluted to a concentration of 30, 3, or 0.3 μm. The solution was incubated for up to 5 h at 37 °C. At the end of the aggregation period, each sample was diluted in serum-free medium to a final dilution of 1:10 (3 μm Aβ_1–42_) and added to the cells. After 72 h of exposure, the cells were visualized for morphological changes and photographed using a Nikon TMS-F inverted phase contrast microscope (Nikon Instruments Inc.) equipped with an Olympus Altra20 camera using the analySIS getIT v.5 software (Olympus Soft Imaging Solutions GmbH). Furthermore, cell viability was determined using the Cell viability assay Kit II (XTT assay; Roche Diagnostics GmbH) according to the manufacturer's instructions. The absorbance at 450 nm and 750 nm was measured after 16 h using a Victor3V 1420 multilabel reader (PerkinElmer). As controls, serum-free medium with 5% (*v*/*v*) diluents or serum-free medium with 5% (*v*/*v*) diluents with 30, 3, or 0.3 μm p-FTAA were used.

##### Aβ Aggregation Kinetics

Aβ_1–42_ was diluted to a concentration of 30 μm in PBS and added to a 96-well microtiter plate (Corning Inc. Life Sciences). The stock solutions of p-FTAA (1.5 mm) and ThT (2 mm) were diluted in distilled water to 15 μm and placed in the wells, leading to a concentration of 3 μm. Samples were incubated at 37 °C in a Tecan Saphire2 microplate reader and the emission spectrum was measured every 20–30 min. The emission spectra for p-FTAA and ThT were collected between 480 and 650 nm, with an excitation wavelength of 440 nm.

##### Analysis of Soluble and Insoluble Aβ Species

Aβ_1–42_ fibrils were prepared by incubating 30 μm peptide alone or with 3 μm p-FTAA in PBS at 37 °C for 0–24 h. The samples were centrifuged at 16,500 × *g* for 10 min, and 5 μl of the supernatants and pellets were collected and vacuum dried, dissolved in HFIP, and lyophilized. The samples were then dissolved in 2 mm NaOH and spotted onto a 0.2 μm nitrocellulose membrane (Bio-Rad). The membrane was blocked in 5% nonfat dry milk in Tris-buffered saline solution with 0.1% Tween (TBS-T; Medicago AB) for 1 h at room temperature and probed with the Aβ antibody 6E10, (mouse monoclonal, 1:1000, Covance, SIG-39300) overnight at 4 °C. For the detection of Aβ amyloid fibrils, Aβ_1–42_ (30 μm) was aggregated alone or with 3 μm p-FTAA in PBS at 37 °C for 0–3 h. The samples were processed and analyzed as described above. The membrane was probed with the anti-amyloid fibrils OC antibody (a generous gift from Dr. Charles Glabe, 1:5000) overnight at 4 °C. The secondary antibodies were horseradish peroxidase-conjugated (Dako) and added for 1 h at room temperature. The dot blots were visualized using Amersham Biosciences™ ECL™ detection systems (GE Health Care).

##### Electron Microscopy

For transmission electron microscopy (TEM) analysis, 10 μl from the pellet samples of Aβ_1–42_ aggregated with or without p-FTAA for 3 h were placed onto carbon-coated copper grids and incubated for 1 min. After removing excess liquid, grids were washed two times with deionized water prior to negatively staining with 2% uranyl acetate for 1 min. Samples were then analyzed with a Jeol JEM-1230-EX electron microscope (Akishima).

##### Circular Dichroism

Circular dichroism (CD) measurements were performed on a Chirascan spectrophotometer (Applied Photophysics) using a 1.0 mm cuvette. Measurements were carried out with an Aβ_1–42_ concentration of 30 μm and p-FTAA concentration of 3 μm in 50 mm phosphate buffer. Spectra (190–250 nm) were recorded at 37 °C every 10 min until no change in the content of soluble β-sheet was observed.

##### Proteolysis of Aβ Fibrils

Aβ_1–42_ fibrils were prepared by incubating 30 μm peptide alone or in the presence of 3 μm p-FTAA in PBS at 37 °C for 24 h, and subsequently digested with 1.4 mg/ml proteinase K (Sigma Aldrich) at 37 °C. After incubation for selected time points (0–7 h), 3 μl of the samples were spotted onto a 0.2 μm nitrocellulose membrane (Bio-Rad) and analyzed by dot blot, as described above. Aβ samples retained on the membrane were detected using the 6E10 antibody. Control experiments confirmed that p-FTAA did not affect the activity of proteinase K.

##### Guanidine-HCl Treatment and ANS Fluorescence

30 μm Aβ_1–42_ with or without p-FTAA (3 μm) was aggregated for 24 h and incubated with 1–6 M guanidine-HCl (Gdn-HCl) for 1 h at 37 °C. Then, 4,4-bis (1-anilinonaphthalene 8-sulfonate) (ANS) was added to the samples at a final concentration of 100 μm. The fluorescence emission spectra of ANS were collected at wavelengths ranging from 470 to 550 nm with an excitation wavelength of 375 nm. The highest peak in fluorescence at 475 nm was used to create the graph.

##### Hydrophobicity Assay

Aβ_1–42_ was dissolved in 20 mm of NaOH to 222 μm and further diluted to 20 μm in PBS with 100 μm ANS with or without 3 μm p-FTAA, and added to a 96-well microtiter plate (Corning Inc. Life Sciences). Samples were incubated at 37 °C in a Tecan Saphire2 microplate reader for 48 h with shaking for 1 s every 10 min, and the emission was measured every 10 min at 450 nm with an excitation wavelength of 375 nm.

##### Seeding Assay

30 μm Aβ_1–42_ with or without p-FTAA (3 μm) was aggregated for 24 h, the fibrils formed were washed twice in PBS and sonicated for 5 min. Sonicated fibrils (seeds) were added to 30 μm Aβ_1–42_ at 1 or 5% concentration before Aβ_1–42_ kinetics was employed. The ThT fluorescence was monitored for 16 h as described above.

##### Quenching/Binding Displacement Assay

Aβ fibrils were formed in the presence or absence of p-FTAA. The fibrils were then washed to remove loosely bound p-FTAA before adding ANS. The fluorescence emission spectra of ANS were collected at wavelengths ranging from 400 to 600 nm with an excitation wavelength of 375 nm. The emission spectra of p-FTAA were collected at wavelengths ranging from 450 to 600 nm with an excitation wavelength of 375 nm. The data were collected using a Tecan Saphire2 microplate reader.

##### Statistical Analysis

All data illustrated in bar graphs are presented as mean and S.D. The minimum number of biological replicates for each data set is indicated by “n” in the figure legends. Unpaired, two-tailed t-tests were used to judge statistical significance. Statistical analyses were created using GraphPad Prism 5 (GraphPad software Inc.).

## Results

### 

#### 

##### p-FTAA Reduces the Toxicity of Aβ

**FIGURE 2.**
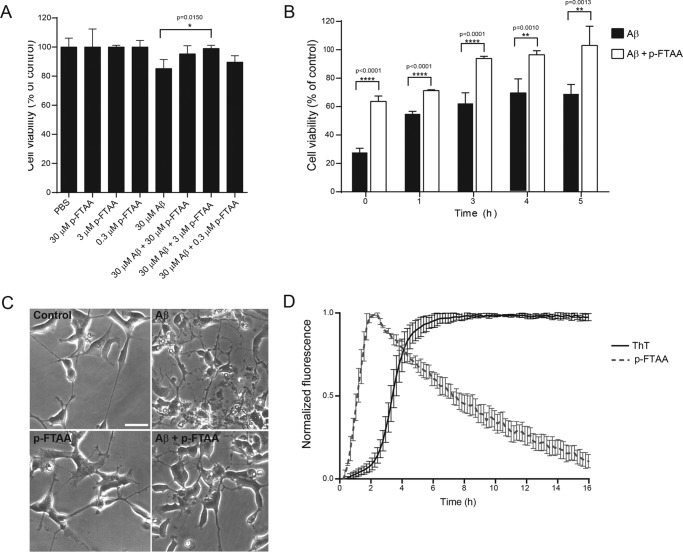
**p-FTAA prevents Aβ toxicity.** *A*, 30 μm Aβ was aggregated alone or with p-FTAA (0.3, 3, and 30 μm) for 1 h, diluted ten times in cell culture medium and added to SH-SY5Y neuroblastoma cells. Cell viability was analyzed 72 h after exposure of the cells to Aβ aggregated alone or with p-FTAA using the XTT viability assay, *n* = 3. *B*, Aβ was aggregated alone or with p-FTAA (3 μm) for different times, diluted ten times and added to cells. Viability is expressed as the percentage of controls, *n* = 3. The bars represent mean ± S.D.; **** *p* ≤ 0.001; ** *p* ≤ 0.01. *C*, phase contrast images of cells after exposure to 30 μm Aβ aggregated for 1 h with or without 3 μm p-FTAA were taken after 72 h of cell exposure. Scale bar = 50 μm. *D*, aggregation kinetics of 30 μm Aβ monitored by fluorescence from 3 μm ThT or 3 μm p-FTAA, *n* = 3.

It was previously demonstrated that p-FTAA is able to bind early formed prefibrillar Aβ species (16, 17) and that these species possess cytotoxic activities (32). Therefore, we decided to investigate whether the binding of p-FTAA could influence the toxicity of these prefibrillar Aβ species. Aβ (30 μm) was aggregated with or without various concentrations of p-FTAA (30, 3, or 0.3 μm) for 1 h. The samples were diluted 10 times, and human neuroblastoma cells were exposed to the samples for 72 h. Aβ alone induced cell toxicity, but co-aggregation with 3 μm p-FTAA increased the cell survival significantly, whereas 0.3 μm p-FTAA did not have a rescuing effect (Fig. 2*A*). For the rest of the experiments, 3 μm p-FTAA was used as the ideal concentration.

To elucidate the capacity of p-FTAA to rescue cells from Aβ toxicity over time, 30 μm Aβ was aggregated with or without p-FTAA for 0, 1, 3, 4, and 5 h. The samples were then diluted 10 times and added to cells. As measured with the XTT viability assay, after 72 h of cell exposure, Aβ was toxic at all times investigated during the Aβ aggregation (Fig. 2*B*). The highest toxicity was detected at 0 h, thus revealing that the toxic effect from Aβ was exerted by aggregates formed initially in the aggregation process. When Aβ was aggregated in the presence of p-FTAA, the toxicity was significantly reduced at all times (Fig. 2*B*). As shown by cell morphology analysis, neuroblastoma cells that were exposed to Aβ lost their neuronal morphology and appeared shrunken (Fig. 2*C*). When cells were exposed to Aβ that were co-aggregated with p-FTAA, the morphological changes, such as the breakdown of cell processes and the appearance of shrunken cell bodies, were less evident. No change in cell morphology was observed when the cells were treated with vehicle or only p-FTAA (Fig. 2*C*). Next, the aggregation process of 30 μm Aβ was monitored with fluorescence from 3 μm p-FTAA or 3 μm ThT (Fig. 2*D*). The p-FTAA signal did not show any lag phase and the elongation phase, which increased more rapidly than the ThT signal, peaked at 2 h of aggregation, and was followed by a decrease in the signal at the later stages of the fibrillation kinetics. On the contrary, the ThT fluorescence signal had a 2 h lag phase, followed by an increase in the signal that peaked at 5 h of aggregation and then reached a steady state plateau that remained constant throughout the experiment. These data show that p-FTAA was able to bind and detect Aβ species that were formed early in the aggregation process. Combined with the cell study, which revealed the highest cell toxicity from early formed Aβ species, these findings point toward that p-FTAA elicits its protective effect via binding to these early formed Aβ species and thereby rescues the cells from Aβ toxicity.

##### p-FTAA Increases the Proportion of Insoluble Aβ Species

**FIGURE 3.**
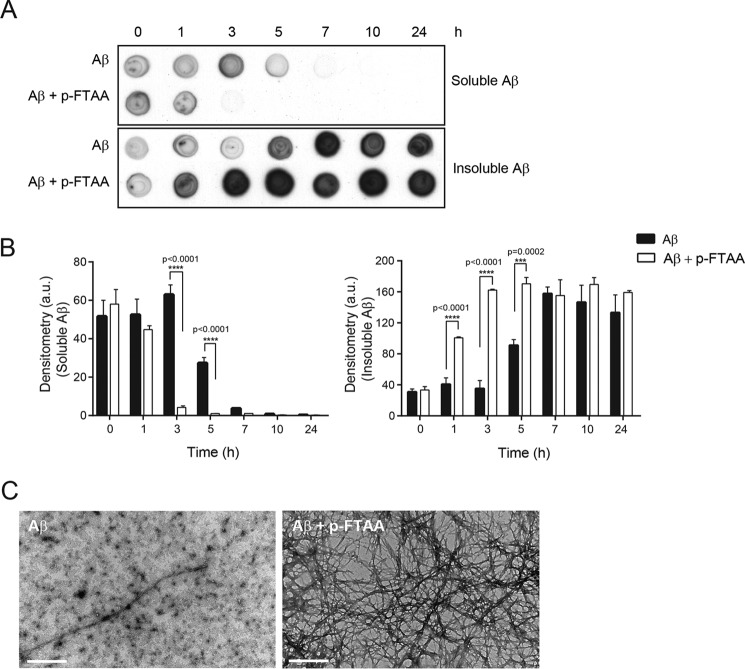
**p-FTAA changes the ratio between soluble and insoluble Aβ species.** 30 μm Aβ was aggregated alone or with 3 μm p-FTAA. *A*, dot blot analysis of supernatants (*Soluble A*β) and pellets (*Insoluble A*β) from Aβ aggregated alone or with p-FTAA for different times by using the 6E10 antibody. The dot blot is one representative experiment out of three. *B*, densitometric quantification of the dot blots in *A, n* = 3. *C*, transmission electron microscopy images of pellets from Aβ aggregated alone or with p-FTAA for 3 h, scale bar, 1 μm.

To investigate whether p-FTAA could affect the equilibrium between soluble and insoluble Aβ species during the aggregation process, Aβ was aggregated alone or with p-FTAA for 0, 1, 3, 5, 7, 10, and 24 h. At each time point, samples were centrifuged and the supernatants, which contained the soluble Aβ species, and the pellets, containing insoluble Aβ aggregates, were collected. Dot blot analysis revealed that in the presence of p-FTAA, the level of soluble Aβ species started to disappear after 1 h and was decreased significantly after 3 h and that, at the same time frame, insoluble Aβ aggregates significantly appeared (Fig. 3, *A* and *B*). When Aβ was aggregated alone, the soluble species disappeared after 5 h and insoluble Aβ aggregates appeared at a distinct level in the pellet after 5 h of aggregation. Pellets from Aβ samples that were aggregated for 3 h with and without p-FTAA were analyzed by TEM. Aβ aggregated alone showed a mixture of globular, prefibrillar, and fibrillar structures, whereas Aβ co-aggregated with p-FTAA contained primarily dense fibrillar structures (Fig. 3*C*). Our results demonstrate that p-FTAA was able to shift the equilibrium between soluble and insoluble Aβ species toward formation of insoluble Aβ fibrils. Thus, the rescue effect mediated by p-FTAA seems to be caused by the ability of p-FTAA to reduce the population of soluble toxic Aβ species by promoting formation of insoluble Aβ fibrillar aggregates.

##### p-FTAA Promotes the Formation of β-Sheet-rich Structures of Aβ, Which Are Degradation-resistant

**FIGURE 4.**
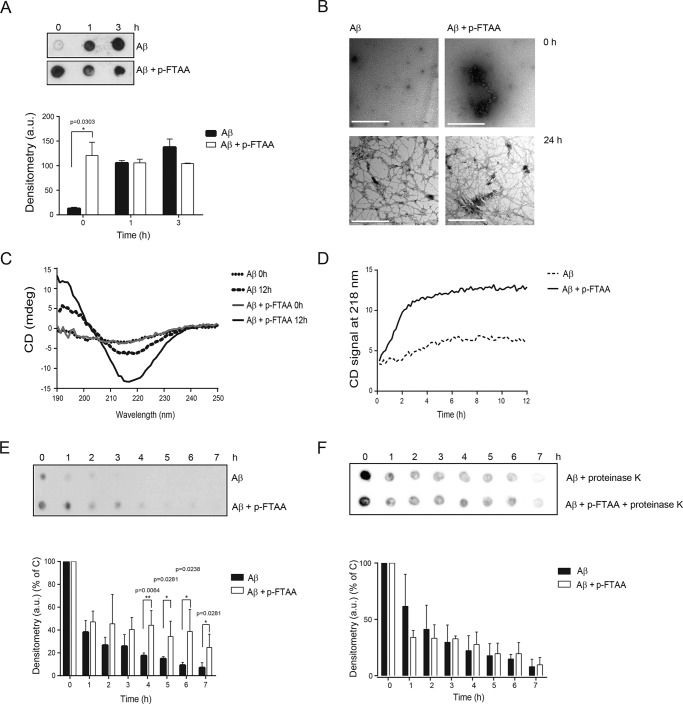
**p-FTAA promotes Aβ fibrillogenesis and proteolytic degradation resistance.** 30 μm Aβ was aggregated alone or with 3 μm p-FTAA. *A*, dot blot analysis of Aβ aggregated alone or with p-FTAA for different times using the fibril specific OC antibody. The dot blot is one representative experiment out of three biological replicates. Densitometric quantification of the three dot blots is also shown. *B*, transmission electron microscopy images of Aβ aggregated alone or with p-FTAA at 0 and 24 h, scale bars, 0.5 μm. *C*, circular dichroism analysis of Aβ fibrillation for 0 and 12 h with or without p-FTAA. The graph represents one experiment out of three. *D*, circular dichroism at 218 nm during aggregating of Aβ with or without p-FTAA for 12 h. The graph represents one experiment out of three. *E*, proteolytic degradation analysis of Aβ fibrils formed with or without p-FTAA. Samples were aggregated for 24 h at 37 °C and then digested with proteinase K for the indicated times at 37 °C. *F*, proteolytic degradation analysis of Aβ fibrils when digested with proteinase K in the absence or presence of p-FTAA. The *E* and *F* results were analyzed with dot blot using the 6E10 antibody, and the dot blots are a representative experiment out of three biological replicates. Densitometric quantification of the three dot blots is also shown.

To further explore the effect of p-FTAA on Aβ fibril formation, Aβ was aggregated with or without p-FTAA, and samples were collected after 0, 1, and 3 h for dot blot analysis using the OC antibody, which specifically recognizes amyloid fibrils (33). Already at the start of the aggregation process (0 h), Aβ aggregated with p-FTAA formed OC positive fibrils, as revealed by dense dots (Fig. 4*A*). However, when Aβ was aggregated alone, the OC antibody only faintly recognized Aβ fibrils, demonstrated as a significant lower detection level of fibrils by OC (Fig. 4*A*). At 1 and 3 h of Aβ aggregation, OC staining was detected for Aβ samples aggregated either alone or with p-FTAA (Fig. 4*A*). The 0 h samples were analyzed with TEM. Aβ aggregated alone showed globular-shaped structures with the presence of small prefibrillar aggregates, whereas Aβ aggregated with p-FTAA contained fibrils as well as globular-shaped structures (Fig. 4*B*).

To monitor the formation of the secondary structure of Aβ during the aggregation process, CD analysis was performed. At 0 h, the CD spectra indicated smaller amounts of mainly β-sheet secondary structure in the presence of p-FTAA, which is in accordance with the OC signal in the dot blot at 0 h (Fig. 4, *A* and *C*). At 12 h of aggregation, the CD spectra obtained for Aβ aggregates that were formed with or without p-FTAA displayed a minimum at 218 nm, which indicates β-sheet formation, where the negative peak was more pronounced for the sample with p-FTAA (Fig. 4*C*). To monitor the formation of β-sheet structure over time, the CD signal at 218 nm was recorded every 10 min during 12 h. This result revealed that the β-sheet formation peaked at 3 h for Aβ aggregated with p-FTAA and at 6 h for Aβ aggregated alone (Fig. 4*D*). Thus, the formation of β-sheet structures occurred faster when Aβ was aggregated in the presence of p-FTAA than when it was aggregated alone. These results further strengthen our hypothesis that the rescue effect exerted by p-FTAA on Aβ toxicity was due to the capacity of p-FTAA to bind soluble toxic Aβ species and promote formation of non-toxic amyloid fibrils.

TEM pictures captured after aggregation of Aβ with or without pFTAA for 24 h revealed formation of well-structured Aβ fibrils with similar morphologies both in the presence and in the absence of p-FTAA (Fig. 4*B*). To investigate the resistance of these fibrils toward proteolytic degradation the formed amyloid fibrils were incubated with proteinase K for up to 7 h. Dot blot analysis demonstrated that Aβ fibrils formed without p-FTAA were degraded significantly after 4 h, whereas Aβ fibrils formed with p-FTAA were still detected at 7 h of incubation with proteinase K (Fig. 4*E*). p-FTAA did not inhibit the activity of proteinase K during the proteolytic degradation assay (Fig. 4*F*). Taken together, these results showed that p-FTAA is able to trigger a change in the Aβ aggregation pathway that results in rapid formation of fibrillary Aβ that are rich in β-sheet structure and possess an increased resistance to proteolytic degradation.

##### p-FTAA Reduces the Hydrophobicity of Aβ Fibrils

**FIGURE 5.**
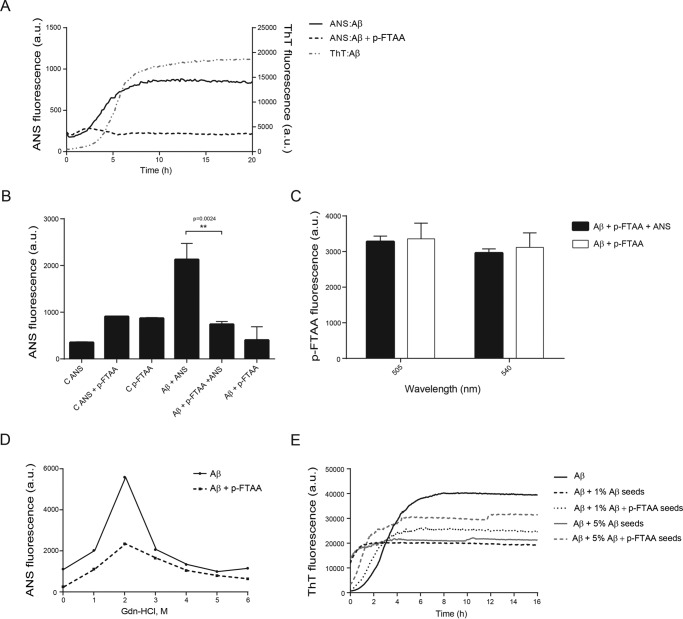
**p-FTAA protects Aβ fibrils from exposure of hydrophobic surfaces.** *A*, analysis of the hydrophobicity of Aβ alone or with p-FTAA during the aggregation process using ANS emission at 450 nm in parallel with ThT emission at 480 nm. The ANS signal from the controls (ANS and ANS+p-FTAA) has been subtracted from ANS:Aβ and ANS:Aβ+p-FTAA curves, respectively. The ThT signal from the control has been subtracted from the ThT:Aβ. The graph is representative of one experiment out of three biological replicates. *B*, ANS fluorescence at 475 nm of washed Aβ fibrils formed in the presence or absence of p-FTAA with and without addition of ANS. *C*, p-FTAA fluorescence at 505 and 540 nm, with the excitation wavelength 375 nm, of washed Aβ fibrils formed in the presence of p-FTAA with and without addition of ANS. *D*, analysis of the exposure of the hydrophobic residues of Aβ fibrils formed with or without p-FTAA after denaturation with different concentrations of Gdn-HCl (1–6 m) using ANS fluorescence at 475 nm. The graph represents one experiment out of three. *E*, ThT analysis of the seeding effect of 1 and 5% fibrillar Aβ seeds formed with or without p-FTAA on Aβ aggregation. The graph represents one experiment out of three biological replicates.

To obtain information whether p-FTAA could have any influence on the hydrophobicity of Aβ aggregates, the aggregation process was monitored with ANS fluorescence, in the presence or absence of p-FTAA, in parallel with ThT fluorescence (Fig. 5*A*). For Aβ aggregated without p-FTAA, the ANS signal followed a sigmoidal curve and reached a steady state phase after ∼8 h. The increase in the ThT signal did also show a sigmoidal curve, but with twice as long lag phase as ANS. In contrast, the ANS signal for Aβ aggregated in the presence of p-FTAA reached a steady state phase after 3 h of aggregation, where the maximum intensity was ∼40% lower compared with the maximum intensity of the ANS signal without p-FTAA.

To test for fluorescence quenching or binding displacement effects between ANS and p-FTAA, Aβ fibrils were formed in the presence or absence of p-FTAA. The fibrils were then washed to remove loosely bound p-FTAA before adding ANS. Fig. 5*B* shows the ANS fluorescence recorded at 475 nm. A clear increase in the fluorescence signal was detected when ANS was added to the Aβ fibrils formed without p-FTAA compared with controls. This signal was significantly reduced when ANS was added to Aβ fibrils formed in the presence of p-FTAA. To examine if the reduced ANS fluorescence could be due to quenching of the signal by p-FTAA, the emission from p-FTAA was examined at 505 nm and 540 nm (the wavelengths that show the maximum emission intensity for fibril bound p-FTAA) using the excitation wavelength for ANS (375 nm). No significant differences in the p-FTAA fluorescence were detected between Aβ aggregated with p-FTAA with no addition of ANS, and Aβ aggregated with p-FTAA followed by addition of ANS (Fig. 5*C*). This result rules out that the ANS fluorescence can be quenched by p-FTAA because if so, the p-FTAA signals of the sample where ANS was added should have higher intensities than the p-FTAA signals of the sample without ANS. To further address differences in the exposure of hydrophobic surface of the fibrils, ANS binding analysis was performed at various concentrations of Gdn-HCl (1–6 m). As shown in Fig. 5*D*, the concentration of 2 m Gdn-HCl caused a change in the Aβ fibrillar structure formed both with and without p-FTAA, which resulted in exposure of hydrophobic surfaces as demonstrated by a significant increase in ANS fluorescence. Aβ fibrils formed in the presence of p-FTAA had lower ANS fluorescence at 2 m Gdn-HCl than did Aβ fibrils formed alone. In summary, these results points toward that p-FTAA was able to reduce the amount of exposed hydrophobic patches available for ANS binding, on the aggregated Aβ structures, which demonstrates that p-FTAA rendered Aβ fibrils less prone to expose hydrophobic regions.

The propagating properties of Aβ fibrils formed in the presence of p-FTAA were investigated by using Aβ fibril seeds formed in the presence or absence of p-FTAA. The seeds were washed to remove any excess of p-FTAA and sonicated. 1 and 5% seeds of Aβ resulted in a faster aggregation detected using fluorescence, but when p-FTAA was present in the seeds the aggregation propagation was attenuated (Fig. 5*E*).

## Discussion

In this study, we demonstrate that the toxicity of the Aβ peptide was greatly reduced when p-FTAA was present during the aggregation process. In addition, soluble Aβ species were more rapidly converted into insoluble amyloid fibrils when Aβ was aggregated in the presence of p-FTAA. More detailed studies on the influence of p-FTAA on Aβ fibril formation showed that p-FTAA promoted the formation of β-sheet rich amyloid fibrils with a higher resistance toward proteolytic degradation and a lower ability to expose hydrophobic residues during denaturing conditions.

Our data show that p-FTAA rescued neuroblastoma cells from Aβ-mediated toxicity by reducing the pool of toxic Aβ species. This protective effect was noticed directly after the Aβ aggregation process started, which implies that p-FTAA was rapid in exerting its rescuing effect on Aβ toxicity. This effect was also long-lasting because at all of the aggregation times tested, where Aβ was toxic, the presence of p-FTAA had a rescuing effect. Fibrillation kinetics revealed differences in the fluorescence profiles of p-FTAA and ThT. When Aβ was aggregated with ThT the fluorescence profile showed a lag phase, whereas no lag phase was detected in the kinetic profile when Aβ was aggregated with p-FTAA. As revealed by the cell experiments, the highest Aβ toxicity was found at the beginning of the lag phase. Thus, the capacity of p-FTAA to rescue Aβ toxicity seems to be due to the ability of p-FTAA to bind to toxic Aβ species that are formed in the lag phase, thereby preventing their toxic action. The lag phase is a critical step in the fibrillation process, where small oligomeric species are formed that act as seeds for the elongation process. The Aβ fibrillation kinetics in the presence of p-FTAA showed that the fluorescence peak coincided with the transition between the end of the lag phase and the beginning of the elongation phase when Aβ was aggregated with ThT. This result clearly reveals that p-FTAA-positive species are formed faster compared with ThT-positive species, which indicates that p-FTAA plays a key role in defining the nucleation kinetics. In fact, p-FTAA could have the capacity to reduce the population of toxic Aβ species formed in the lag phase by promoting formation of non-toxic p-FTAA positive species. The fluorescence of p-FTAA dropped immediately after reaching its highest level, whereas the ThT fluorescence remained high after reaching its maximum. This may indicate that p-FTAA positive fibrils are more prone to precipitate than ThT positive fibrils and/or that the p-FTAA signal is quenched within these fibrils.

To explain how p-FTAA rescued cells from Aβ toxicity, we investigated whether p-FTAA could change the pattern of formation of soluble *versus* insoluble Aβ species. The separation of Aβ species aggregated with p-FTAA demonstrated a reduced presence of soluble Aβ species and increased levels of insoluble Aβ aggregates compared with Aβ aggregated alone, as demonstrated by dot blot. In addition, the TEM data showed a higher content of insoluble Aβ species with fibrillar structures formed after 3 h in the presence of p-FTAA compared with the Aβ sample without p-FTAA. These results suggest that p-FTAA enhanced the formation of insoluble Aβ aggregates with fibrillar structures. To further investigate this phenomenon, we took advantage of the OC antibody, which is a conformation-dependent fibril specific antibody (33). At the starting point of the aggregation process, the OC antibody clearly detected the presence of fibrils when Aβ was aggregated with p-FTAA, whereas only a low signal from the OC antibody was detected when Aβ was aggregated alone. Aβ seeds are the first aggregates that appear in the fibrillation process and are considered the inducers of Aβ deposition. According to Seilheimer *et al.* (34), seeds have globular appearance and are the first detectable structures with TEM. Our data demonstrated that both fibrillar and globular structures were detected at the starting point of Aβ aggregation in the presence of p-FTAA, whereas only globular structures occurred when Aβ was aggregated alone. This result suggests that p-FTAA has the ability to rapidly convert globular seeds to Aβ fibrillar structures. CD analysis showed that during the aggregation process, Aβ adopted an average conformation that was rich in β-sheets. The presence of p-FTAA accelerated the shift from random coil structures toward ordered β-sheets, which indicates that p-FTAA increased the rate of β-sheet formation of Aβ. Therefore, our hypothesis is that by binding to early-formed toxic Aβ species and accelerating the formation of insoluble amyloid Aβ fibrils, p-FTAA is able to greatly reduce Aβ toxicity.

AD is the most common age-related disorder, and because the number of people suffering from this disease is expected to rise dramatically, the development of new compounds to treat AD is in high demand. One therapeutic intervention strategy is based on altering the amyloid aggregation pathway to reduce the content of toxic Aβ species. Previous studies have shown that the compounds orcein-related O4 and curcumin drive the formation of Aβ amyloid fibrils and thereby reduce Aβ toxicity (27, 28). p-FTAA seems to have a similar mechanism of action because it promoted the formation of insoluble Aβ fibrils and decreased the concentration of soluble Aβ species, which are likely responsible for cell toxicity. Earlier, it was demonstrated that p-FTAA stains Aβ deposits in brain tissue from AD transgenic mice and passes the blood brain barrier (BBB) in aged APP/PS1 mice (16). The combined properties of crossing the BBB and decreasing the toxicity of Aβ make p-FTAA a therapeutic candidate for AD.

It has previously been published that exposure of hydrophobic residues on Aβ aggregates highly correlates with a certain degree of cellular toxicity (35), and Campioni *et al.* (36) showed that the toxicity can be reduced if the toxic oligomers are tightly packed with their hydrophobic residues incorporated in the interior of the protein. To assess the hydrophobicity of Aβ with or without p-FTAA we took advantage of the ANS probe whose fluorescence is dependent on the environment and exposure of hydrophobic amino acids (37). The buildup of more hydrophobic surfaces on Aβ aggregated alone compared with Aβ aggregated with p-FTAA indicates that p-FTAA rendered Aβ less prone to expose its hydrophobic residues during the aggregation process. However, in our study high ANS fluorescence did not correspond to high toxicity, since the peak in ANS fluorescence occurred at later time points during the aggregation process when formed Aβ aggregates were less toxic to the cells compared with early formed Aβ species. Thus, besides hydrophobic properties, the size of the formed Aβ oligomers is of importance for Aβ toxicity, where small hydrophobic species are more toxic than larger hydrophobic species (32). The cell toxicity data showed that the highest toxic effect of the Aβ peptide occurred at the start of the aggregation process (0 h) and already after 1 h the Aβ peptide was less toxic. Dot blot analysis using the OC antibody showed that Aβ without p-FTAA contained less OC positive fibrils at 0 h compared with OC positive fibrils at 1 h. Clearly, the formation of OC positive fibrils correlated with lower Aβ toxicity. In the presence of p-FTAA the Aβ sample showed a high content of OC positive fibrils already at 0 h and at this time the cell toxicity data showed a clear rescue effect from p-FTAA. TEM analysis at 0 h confirmed a more rapid formation of fibrils in the presence of p-FTAA. Hence, we conclude that p-FTAA promotes formation of larger Aβ aggregates, with less toxic properties, and reduces the hydrophobicity of Aβ fibrils.

It is also important to investigate how p-FTAA could influence the stability of well-structured fibrils formed in its presence. Most amyloid fibrils, such as those derived from insulin, β2-microglobulin, lysozyme and Aβ, have a high degree of resistance to denaturants and can be dissolved only with harsh solvent conditions, such as Gdn-HCl (38) or DMSO (39). Our study showed that p-FTAA was able to induce a change in the structural integrity of amyloid fibrils by protecting the Aβ fibrils from proteolysis as well as hydrophobic domain exposure. These results indicate that Aβ aggregated with p-FTAA had a more compact conformation. Thus, p-FTAA influences both the aggregation properties of the Aβ peptide and the conformation features of the amyloid fibrils. In a similar manner, the LCP PTAA was previously shown to increase the compactness of scrapie prion (PrP^sc^) fibers and both PTAA and p-FTAA were demonstrated to enhance the resistance of PrP^sc^ to proteinase K proteolysis. These molecules were also shown to reduce the infectiousness of the prion aggregates (30, 31). Our results demonstrate that Aβ-seeds formed in the presence of p-FTAA have a decreased propensity to propagate Aβ aggregation compared with Aβ-seeds free from p-FTAA. This suggests that p-FTAA has the potential to reduce transmission of Aβ in AD in a similar fashion as p-FTAA reduces the infectiousness of prion aggregates.

To characterize and discriminate pathological hallmarks in neurodegenerative disorders are important to understand the pathogenesis of these diseases. p-FTAA spectrally separates Aβ and tau aggregates in AD brain tissue (16) and stains α-synuclein fibers (40). LCOs have been used to discriminate different prion strains (41, 42) and a combination of different LCOs can monitor age-related changes of Aβ plaques from transgenic mice (43). These properties of LCOs can perhaps be used to develop diagnostic tools for neurodegenerative disorders. However, to develop LCOs as diagnostic tools will require careful investigations to clarify if/how the LCO would affect the target protein aggregates.

The use of therapeutics that promote Aβ aggregation into an insoluble fibrillar structure is controversial since the extent to which the Aβ plaque load affects neurodegeneration and synaptic loss has not been established. It has been suggested that the periphery of Aβ plaques is responsible for axonal dystrophies and synaptic degeneration (44) and that the peripheral area of the plaques is in equilibrium with soluble prefibrillar and monomeric Aβ species, which could be involved in AD pathology (4). Moreover, oligomeric Aβ species can be formed in a secondary nucleation process that requires the surface of fibrils (45). In contrast, healthy individuals with a heavy plaque load can still have normal cognitive functions, which indicate that plaques can also be completely inert with regards to neurodegeneration and synaptic loss (46). A compound that accelerates the fibrillation process might be of therapeutic benefit because it can sequester soluble toxic Aβ species into inert fibrils and promotes fibrillization where the formation of oligomers and protofibrils are bypassed in favor of fibril formation. We demonstrated that p-FTAA induced a conformational stability in the fibrils, which would hypothetically shift the equilibrium between different Aβ species toward plaques.

In summary, this study shows that p-FTAA altered the Aβ fibrillation process; by reducing the content of soluble Aβ species in favor of the formation of fibrillar Aβ aggregates, p-FTAA prevented cytotoxicity. These findings suggest the therapeutic potential of p-FTAA and highlight the importance of exploring the effects of other LCOs on Aβ aggregation and toxicity.

## Author Contributions

Conceived and designed the experiment: L. C., L. S., K. K., A-C. B. Performed the experiments: L. C., L. S., E. N., S. I. K. Wrote the paper: L. C., L. S., K. K., A-C. B. All authors contributed to discussion and the final draft of the manuscript.

## Footnotes

5 The abbreviations used are:

AD: Alzheimer disease
Aβ: amyloid-β
NFTs: neurofibrillary tangles
APP: amyloid precursor protein
LCOs: luminescent-conjugated oligothiophenes
p-FTAA: formyl thiophene acetic acid
h-FTAA: hepta-formylthiophene acetic acid
q-FTAA: quadro-formylthiophene acetic acid
ThT: Thioflavin T.
